# Malodorous biogenic amines in *Escherichia coli*-caused urinary tract infections in women—a metabolomics approach

**DOI:** 10.1038/s41598-020-66662-x

**Published:** 2020-06-16

**Authors:** Scarlett Puebla-Barragan, Justin Renaud, Mark Sumarah, Gregor Reid

**Affiliations:** 1 Centre for Human Microbiome and Probiotics, Lawson Health Research Institute, London, ON Canada; 2 Departments of Microbiology & Immunology and Surgery, University of Western Ontario, London, ON Canada; 3 London Research and Development Centre, Agriculture and Agri-Food Canada, London, ON Canada

**Keywords:** Pathogens, Urinary tract infection

## Abstract

Many women suffer from urinary tract infections (UTIs). In addition to pain and increased urgency to urinate, malodour is a significant issue for these patients. The specific factors causing this malodour are unclear, and there are no targeted treatment options to counteract it effectively. We used a metabolomics approach to compare the chemical composition of metabolites in the urine of women with *E. coli* UTIs (*n *= *15)* and those who are healthy (*n* = 10). The biogenic amines trimethylamine and putrescine, which cause malodour in other urogenital conditions, were significantly increased in UTI patients. Conversely, the precursor of trimethylamine, trimethylamine *N*-oxide, was lower. To further confirm the source of the malodorous compounds, *in vitro* experiments were conducted by incubating strains of uropathogenic *E. coli* in sterilized urine from healthy women. All tested strains accumulated trimethylamine and putrescine. Notably, cadaverine was also produced by *E. coli* strains *in vitro;* however, it was not significantly different between both groups. We confirmed that the malodorous amines TMA and putrescine are found in higher concentrations in the urine of patients with an *E.coli*-caused UTI.

## Introduction

Urinary tract infections (UTIs) are a highly prevalent global health problem, with over 50% of women expected to experience at least one in their lifetime^1,2^. Lower UTIs are more common than those infecting the kidney, and *Escherichia coli* (UPEC) strains are responsible for approximately 80% of the cases. These uropathogens originate from the gastrointestinal tract^3^ and enter the bladder via the fecal-vaginal-periurethral route^4^. These infections are present with dysuria, increased frequency of urination, abdominal pain, and malodour^5^. However, besides ammonia, which is a known cause for foul-smelling urine^6,7^, other sources of malodour during infection and the source of their production require further characterization. None of the currently available treatment regimens target malodour.

A better understanding of the chemical origins of malodour during this condition would enable the creation of more targeted and efficient therapies. Therefore, we aim to identify if known causes of urogenital malodour are also present during UTIs.

**Figure 1:**
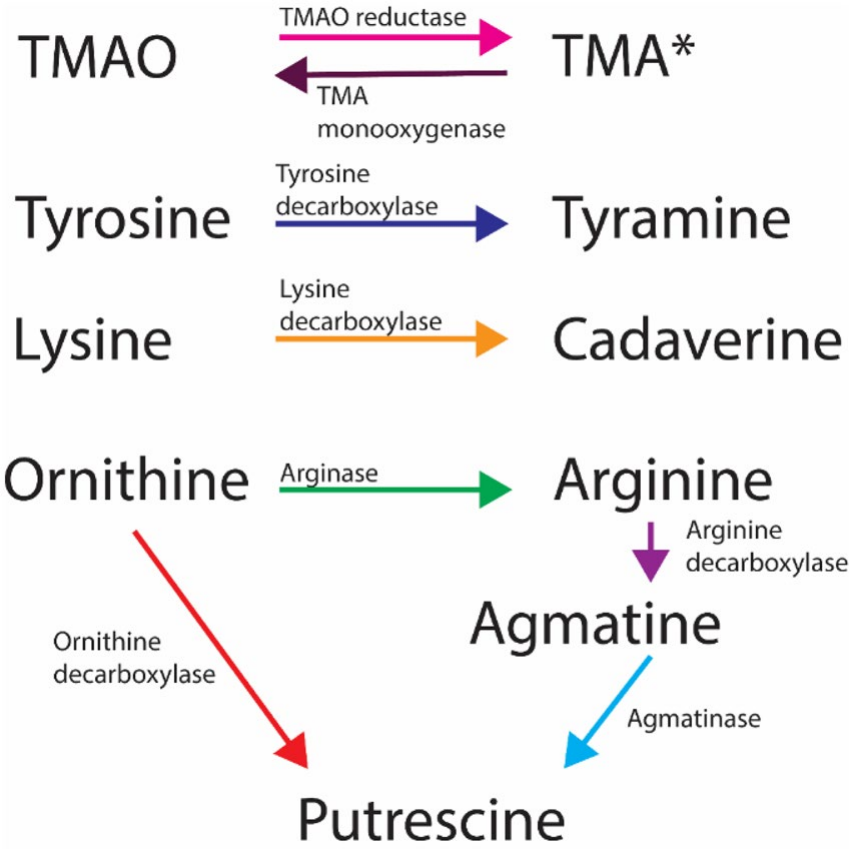
Biosynthetic origin of biogenic amines. Each line represents a single reaction catalyzed by the described enzyme. *Other possible sources for TMA production are described in the text, while the one depicted here is the most relevant in the context of the bladder.

Biogenic amines are basic nitrogenous compounds with well-established organoleptic characteristics (Table 1) associated with malodour^8^. Most of them are biosynthesized from amino acids, as shown in Fig. 1. Trimethylamine (TMA), tyramine, cadaverine, and putrescine are of interest since they are known to cause urogenital malodour in conditions such as bacterial vaginosis (BV)^8,9^. Until now, these compounds have not been associated with urinary malodour, except for trimethylamine (TMA), which has a characteristic fishy odour and has previously been postulated by various groups as a candidate biomarker for *E. coli*-associated UTI^10–12^.

**Table 1:** Odour characteristics of biogenic amines.

Compound	Odour description	Odour detection threshold	Odour strength
Trimethylamine	Fishy/oily/rancid/sweaty/fruity^63^	0.000032 ppm^64^	Very high^63^
Tyramine	Mild/meaty/dirty/cooked/phenolic/rubbery^65^	NA	Moderate^65^
Cadaverine	Sperm/dead-animal/animalic^66^	190 ppm^67^	NA
Putrescine	Animal/rotting/fish^68^	22 ppm^67^	NA

Although the majority of urinary TMA derives from the reduction of trimethylamine *N*-oxide (TMAO) (Fig. 1), other substrates can also yield TMA^8^. Notably, the reverse reaction, where TMA is oxidized to form TMAO is also possible^13^. Other relevant TMA precursors are the products of dietary phosphatidylcholine, choline and betaine^14^, as well as dietary sources such as L-carnitine and ergothioneine^15^. However, these compounds are majorly metabolized into TMA by several taxa of the gut microbiota^16,17^ in the small intestine, transported through the bloodstream to the liver where they are oxidized into TMAO^18^ which subsequently travels to the kidneys to be excreted through the bladder^19^, where it can be converted back into TMA by indigenous or pathogenic bacteria.

Tyramine is synthesized via decarboxylation of tyrosine, and it is produced by uropathogens such as *Enterococcus faecalis*^20^, but it has not been reported in UPEC strains. Cadaverine, which is primarily a bacterial metabolite^21^, is the product of the decarboxylation of lysine, and it is thought to be synthesized by *E.coli* in anaerobic and low pH conditions^22^. Putrescine, produced both by human and bacterial cells^21^, is a product of amino acid catabolism^23^, either from ornithine or agmatine, the latter being a product of the decarboxylation of arginine (Fig. 1).

Evidence suggests these compounds play an essential role in pathogenesis. For instance, entero-invasive strains of *E. coli* are unable to produce cadaverine, a compound that interferes with the invasive process^24^. While putrescine enhances survival within oxidative environments^25^ and augments cell growth and proliferation^26^; consistent with these observations, Satink *et al*.^27^ showed that putrescine levels increased in patients with UTI before antibiotic therapy, then decreased as a result of the treatment, presumably because of the eradication of the producers. However, no differences were detected for cadaverine in either case, likely because urinary cadaverine is primarily derived from the gastrointestinal microbiota and dietary sources^21^.

Additionally, a vaginal strain, *E. coli* 83972, has the genetic machinery to produce putrescine, cadaverine, and TMA. Of note, this strain encodes two enzymes for TMA production^8^, a homolog of TMAO reductase, and choline trimethylamine-lyase, which is the enzyme in charge of transforming choline to TMA, suggesting that, to some extent, TMA could derive from either of these substrates in the urogenital tract. Since the vaginal and bladder microbiomes are interconnected^28^, it cannot be ruled out that this strain can also be present in the bladder. Contrary to common belief, the bladder has its own microbiome^29^, which could have a role in this TMA conversion similarly as it happens in the gut, where TMAO is reduced primarily by *Enterobacteriaceae*^30^, a family of bacteria which is present in larger proportions during a UTI.

It has been reported that cadaverine production increases when UPEC strains are grown under nitrosative stress, potentially conditioning them for enhanced colonization of the host^31,32^. This is of relevance since, during a UTI, there is a significant formation of nitric oxide and reactive nitrogen intermediates^33,34^. Nevertheless, the specific role of cadaverine in resistance to nitrosative stress is unclear. Potentially, during an infection, UPEC strains produce a significant amount of cadaverine, which would, in turn, cause malodorous urine. Yet, previous work has shown no change in cadaverine levels during UTI^27^.

The present study aimed to take a metabolomics approach to identify molecules responsible for urinary malodour and their biosynthetic origins.

## Results

### Clinical sample characterization

In total 31 human urine samples were obtained, comprising both UTI-negative (*n* = 10) and UTI-positive (*n* = 21). Of the latter, *E.coli* was the dominant pathogen in 15 individuals, while *Staphylococcus aureus*, *Klebsiella pneumoniae*, *Pseudomonas aeruginosa* were dominant in 3, 2 and 1 individuals, respectively. There were no significant associations between UTI and urinary pH or creatinine concentration (Supplementary Material, Figs. S1, 2). Only samples positive for *E. coli* will be discussed in the subsequent results. Analyses of samples infected by other pathogens can be found in the Supplementary section.

### Metabolomics of UTI-positive clinical samples

**Figure 2:**
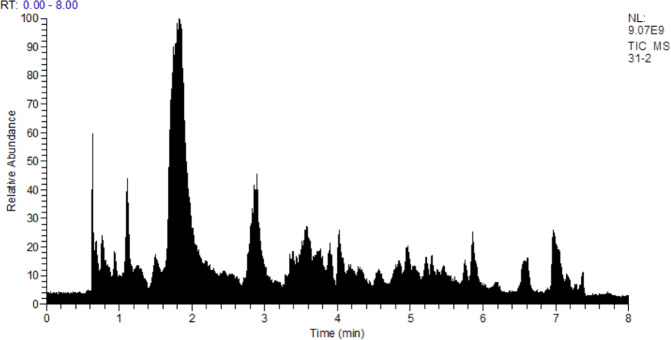
Chromatogram of a UTI positive sample. Separation carried out using HILIC chromatography, ideally suited for polar compounds (e.g. biogenic amines and amino acids).

The metabolomic profiles of the human urine samples were analyzed by high-resolution LC-MS. Biogenic amines and other small polar compounds are not well suited for chromatographic separation using a C18 reverse-phase column. Therefore, a hydrophilic interaction chromatography (HILIC) approach was used (Fig. 2).

**Figure 3:**
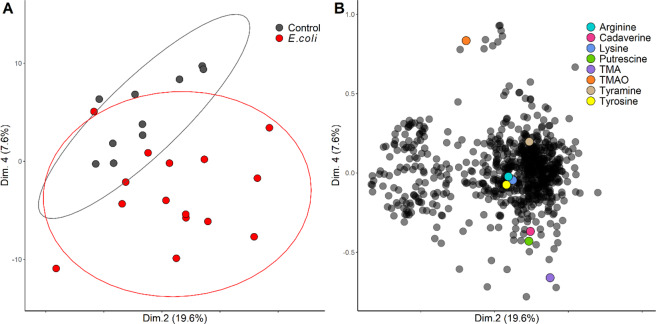
TMA and one of its main precursors, TMAO, are driving the separation between UTI-positive and negative samples in multivariate analysis. (**A**) Principal components analysis (PCA) score plot of Healthy (black, *n* = 10) vs UTI-positive samples (red, *n* = 15), where each point represents a single sample from a single woman. The location of each point displays differences in the metabolome, with samples closer to each other being similar. Ellipses represent the 95% confidence intervals; (**B**) PCA loadings, which show the weights of each metabolite in the principal component cartesian plane. Each point represents a single metabolite. Biogenic amines, as well as their precursors, are colour coded.

The features extracted by XCMS were analyzed using a principal components’ analysis (PCA) model. No distinct grouping of samples could be observed in the 1^st^ dimension. However, separation of both UTI and healthy groups was observed in the 2^nd^ and 4^th^ (27.2% total explained variance). The major chemicals responsible for the separation of these groups were TMA and putrescine (Fig. 3).

**Figure 4:**
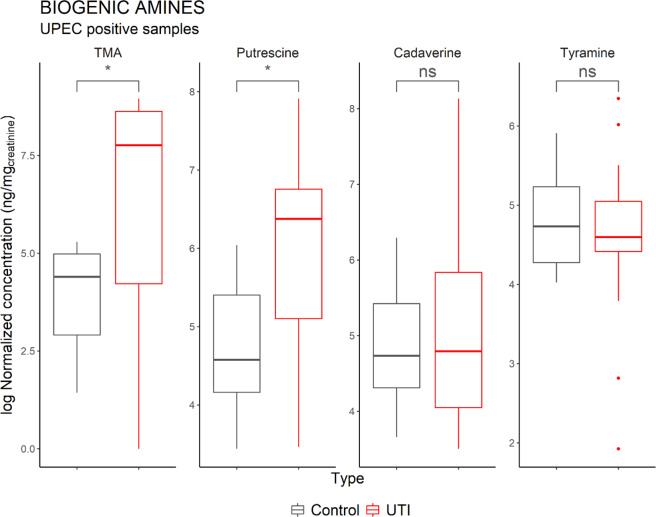
Comparison of the concentration of biogenic amines in Healthy vs UTI positive patients. Control consists of the urine of healthy patients (*n* = 10). TMA and putrescine levels are higher in patients with UPEC caused UTI (*n* = 15). Only the samples positive for UPEC were included in this analysis. Horizontal lines indicate the median. Significant differences were determined on log-transformed values using a two-sample t-test (*p < 0.05).

Biogenic amines, as well as some standard amino acids, were also quantified in the clinical urine samples. Following normalization with urinary creatinine concentrations, both TMA and putrescine were significantly elevated between groups, whereas cadaverine and tyramine were not (Fig. 4).

**Figure 5:**
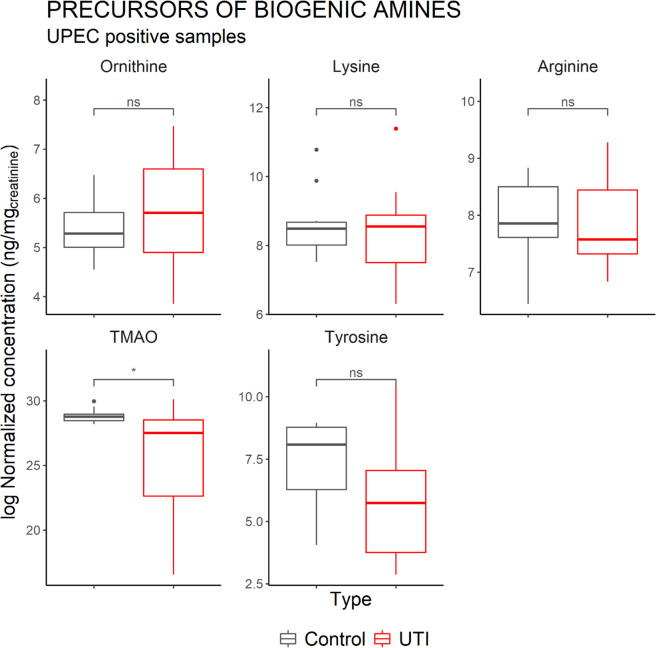
Comparison of the concentration of the precursors of biogenic amines in Healthy vs UTI positive patients. Control consists of the urine of healthy patients (*n* = 10). TMAO, one of the precursors of TMA, was significantly lower in the positive samples. Only the samples positive for UPEC were included in this analysis (*n* = 15). Horizontal lines indicate the median. Significant differences were determined on log-transformed values using a two-sample t-test (*p < 0.05).

**Figure 6:**
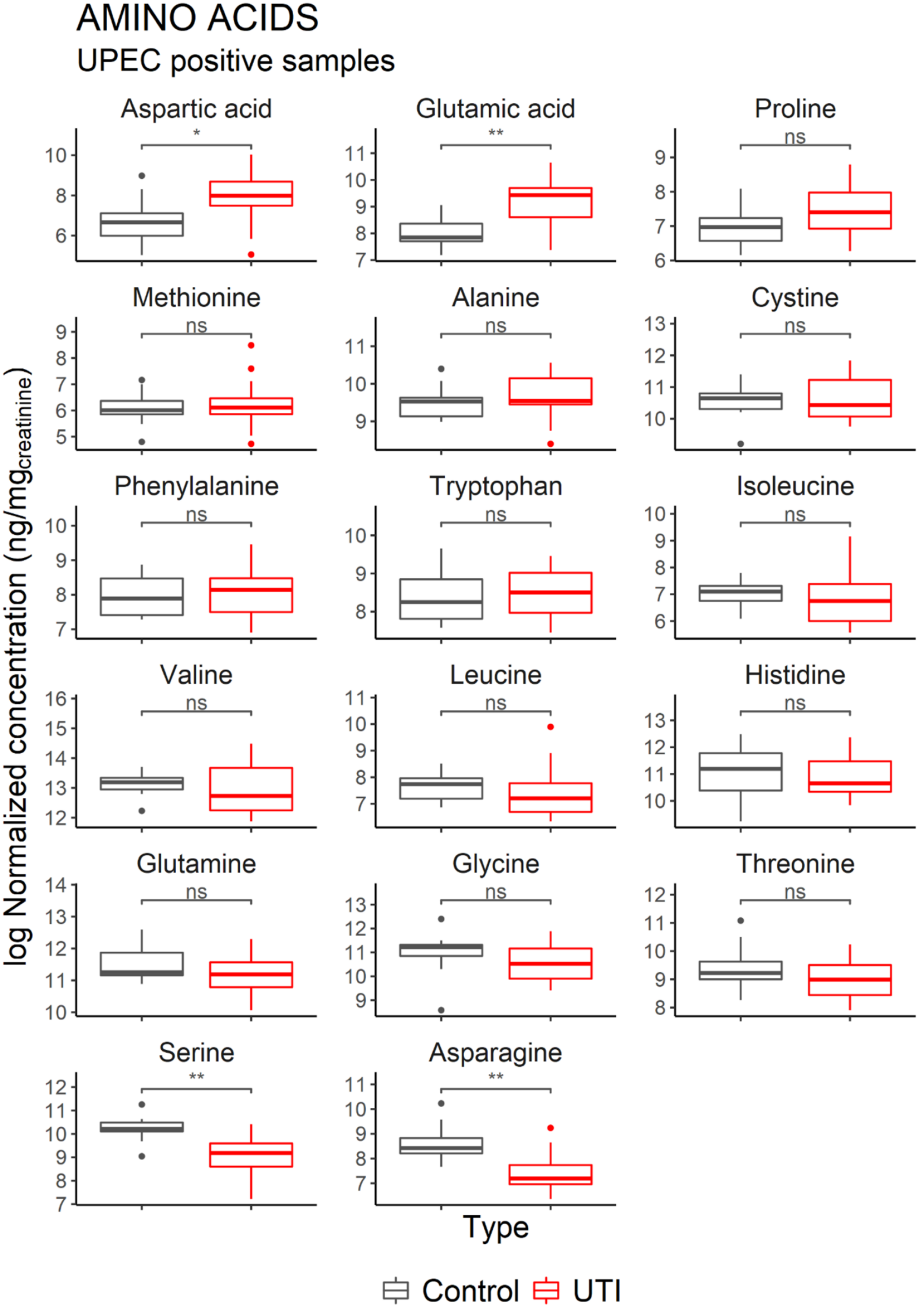
Comparison of the concentration of amino acids in Healthy vs UTI positive patients. Control consists of the urine of healthy patients (*n* = 10). Glutamic acid and Aspartic acid are significantly elevated in UTI patients, while Serine and Asparagine are significantly lower. Only the samples positive for UPEC were included in this analysis (*n* = 15). Horizontal lines indicate the median. Significant differences were determined on log-transformed values using a two-sample t-test (*p < 0.05, **p < 0.005).

The concentrations of the precursors of these amines are shown in Fig. 5; only TMAO—which is one of the possible precursors of TMA—was significantly lower in UTI patients. On the amino acid analysis, aspartic acid and glutamic acid were significantly increased in the UTI positive group, whereas serine and asparagine were decreased (Fig. 6).

### *in vitro* analysis of UPEC strains in sterile urine

**Figure 7:**
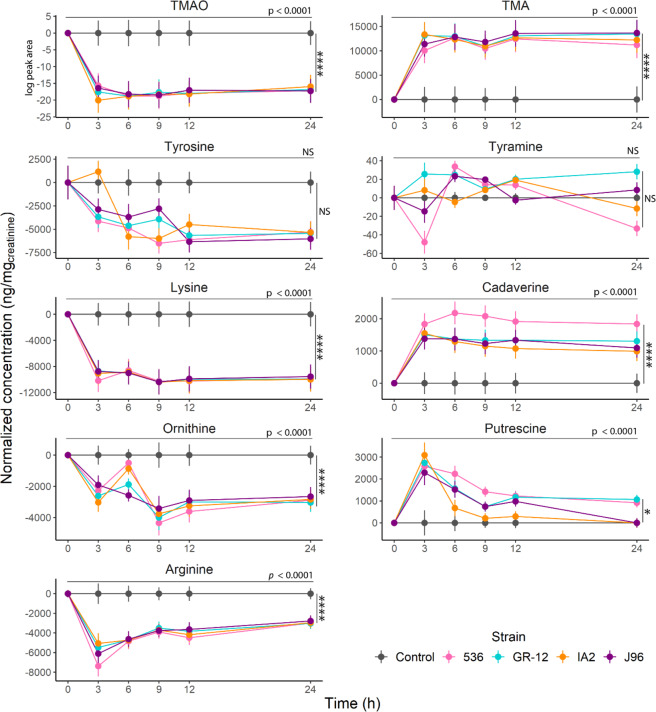
Time course analysis on the formation and uptake of biogenic amines and their precursors by different UPEC strains. Precursors on the left column and biogenic amines on the right. TMAO was analyzed semi-quantitatively based on the log of its peak area. Metabolite levels standardized using the control as a baseline, consisting of sterile urine with 5 µL of LB media to account for potential matrix effect. A linear mixed-effect model with repeated measures was used to calculate statistical significance. Tukey post-hoc test was used to determine individual differences (Tables S1 and S2). The horizontal significance line and p-value correspond to the main effect of Time over the concentration of each metabolite after 24 h. Vertical significance corresponds to the comparison of each strain vs the control. Data are presented as means of 3 independent experiments ± SEM. (*p < 0.05, *****p < 0.0001).

**Figure 8:**
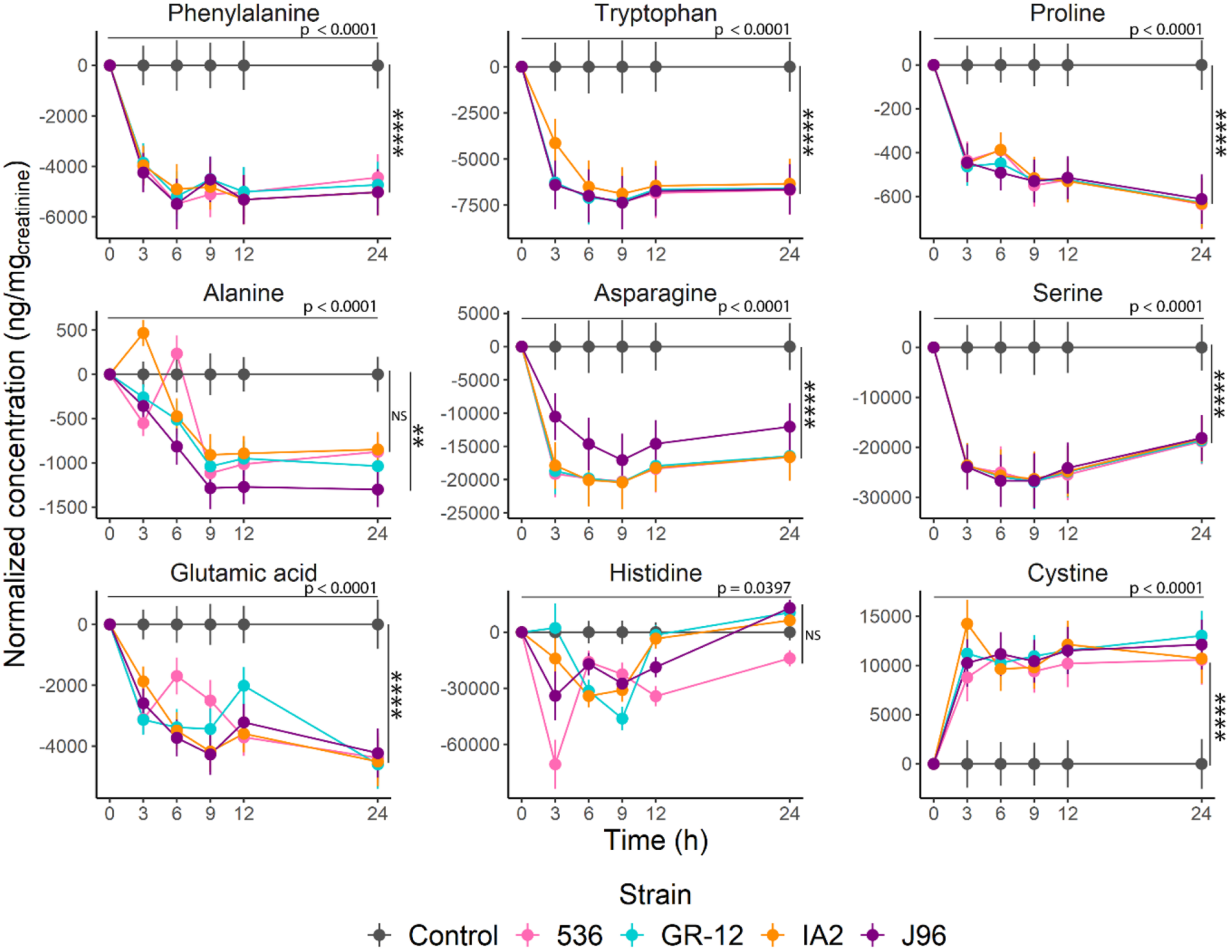
Time-course analysis of the formation and uptake of amino acids by different UPEC strains. Metabolite levels standardized using the control as a baseline, consisting of sterile urine with 5 µL of LB media to account for potential matrix effect. A linear mixed-effect model with repeated measures was used to calculate statistical significance. Tukey post-hoc test was used to determine individual differences (Tables S1 and S2). The horizontal significance line and p-value correspond to the main effect of Time over the concentration of each metabolite. Vertical significance corresponds to the comparison of each strain vs the control after 24 h. Data are presented as means of 3 independent experiments ± SEM. (**p < 0.01, *****p < 0.0001).

To better understand the metabolic dynamics involved in the production of biogenic amines by UPEC, the presence of metabolites from four different clinical UPEC strains grown in sterile female urine was assessed over 24 hours. Many of the biogenic amines identified in clinical samples were also found *in vitro* and showed significant changes within 3 hours (Figs. 7 and 8).

Similar trends in terms of metabolite changes were observed between all five tested UPEC strains (Tables S1 and S2), showing an accumulation of cadaverine and TMA, whereas a maximal amount of putrescine was produced after 3 h and diminished thereafter. Except for cystine, where an accumulation over time was observed, most amino acids were reduced in concentration by the UPEC strains during the incubation. Alanine showed significant increments at hours 3 and 6 by strains IA2 and 536 respectively and was degraded thereafter; after 24 h only strain J96 was significantly different from the control. Histidine and tyramine did not show a major change in amounts relative to their starting concentrations. The amino acids: aspartic acid, methionine, isoleucine, valine, leucine, glutamine, glycine, and threonine were not included in this analysis as they were below the detection limit of the method.

## Discussion

Here, we present a comprehensive profile of the production of odorous compounds during UPEC-UTI. Only the biogenic amines TMA and putrescine were present at high concentrations in patients with UTIs compared to controls. This contrasts with results in patients with BV, where cadaverine and tyramine are also elevated^8,9^.

Using an *in vitro* model, cadaverine was found to be significantly increased, perhaps due to nitrosative stress^33,35^ which is higher in a steady-state culture than in the human bladder, allowing for its accumulation. The *in vitro* model provides a static system that allows for better appreciation of the rates of consumption and production of specific metabolites, while *in vivo*, there is a continuous flow of urine into the bladder from the kidneys that replenishes substrates and makes it challenging to identify the changes in metabolite concentration.

Notably, differences were also found in cadaverine levels between strains. Following reports that cadaverine production can interfere with the invasive process of UPEC, we hypothesize that its production is reduced during colonization of the bladder as a means to enhance infectivity^24^. Thus, the environment of the bladder may influence the expression of the genes that regulate cadaverine production and perhaps explain why previous reports have found contrasting results.

Putrescine levels were increased *in vivo* and *in vitro* in agreement with findings in patients with an overactive bladder^35^. This is associated with an increase in ornithine decarboxylase, which has been shown to block the calcium potassium channel. Since putrescine levels can modulate intracellular calcium levels in urothelial cells, it suggests a correlation between UTIs and overactive bladder^36^ and a role for putrescine in urinary urgency. This was further supported by the finding that ornithine, a major precursor of putrescine, showed a marked decrease in the *in vitro* samples. More studies are warranted to investigate this result.

TMA was one of the metabolites that exhibited a highly significant increase in the present study. Our method detected that TMAO, one of the precursors of TMA, was significantly lower in UTI-positive patients, which suggests that it was one of the main sources of production. However, it cannot be ruled out that, at least part of it, was derived from other dietary sources (i.e. choline, betaine, L-carnitine, ergothioneine). Furthermore, we acknowledge that some of the TMAO observed could be a direct consequence of the increase in TMA. Regardless of this fact, the difference in the concentration of TMAO between both groups is still significant.

Future studies that target other potential precursors should aim to elucidate the proportions of the sources from were TMA is being produced during the course of a UTI. It would be worth investigating whether the increase of TMA during a UTI could affect other symptoms and signs. For instance, acetylcholine present in urine can be converted into choline via the enzyme AChE, allowing TMA to be produced from choline through choline TMA lyase activity. The cholinergic system has an important role in voiding the bladder^36^, with acetylcholine from parasympathetic nerves and non-neuronal cells within the urothelium, affecting, directly and indirectly, muscle contraction^36^.

Except for cystine, most of the amino acids analyzed in our time-course were degraded over time. Previous reports have shown that when UPEC strains are grown in urine, several genes responsible for amino acid catabolism are upregulated^37^. It is thought that this is important in the ability of UPEC to colonize the bladder^38^. Notably, serine was significantly reduced both *in vivo* and *in vitro*, suggesting it is being used as a substrate by UPEC. This is consistent with previous reports showing upregulation of genes responsible for amino acid catabolism, specifically D-serine deaminase (*dsdA*) when UPEC were grown in human urine^37,39^. Both D- and L-serine are gluconeogenic amino acids that can be degraded to produce oxaloacetate or pyruvate, which can enter the TCA cycle, therefore they can be used as a carbon source^38^. Furthermore, the catabolism of D-serine in the urinary tract has been identified as a key signalling mechanism for virulence gene expression^40–42^.

A significant decrease in asparagine levels was also found. Asparagine metabolism by *E. coli* has not been extensively studied, but it is hypothesized that the organism can utilize this amino acid by conversion to aspartic acid using an asparaginase enzyme^43^. This agrees with our results, where asparagine was significantly reduced while aspartic acid increased.

Overall, this study revealed that the malodorous amine putrescine is higher in patients with a UTI, and confirmed previous findings^10–12^ that correlate an increase in the concentration of TMA with this condition, a compound that is well known for its fishy smell. Although malodour was not measured directly, the clear increase in the concentrations of malodorous compounds suggests that they play a significant role in the urinary malodour that patients with UTIs experience. However, we recognize that future odour metrology studies are warranted, where a trained panel could correlate specific increases of these compounds to the changes in the organoleptic properties of the urine of infected patients^18^.

Our findings open the door to the development of more targeted approaches to reduce urinary malodour, a problem that is particularly prevalent in urinary incontinence patients and takes days to resolve when antibiotics are administered. For instance, specific lifestyle modifications could be recommended to the patients, such as dietary changes, consumption of prebiotics or prebiotics in order to modulate the production of these compounds and their precursors; as well as the addition of specific agents that could degrade or neutralize them in hygiene products (such as diapers and pads). Ultimately, the knowledge of the specific compounds to counteract allows for specific procedures to reduce them effectively, without having to wait for antibiotics to provide some relief.

## Materials and Methods

### Urine samples collection and processing

**Figure 9:**
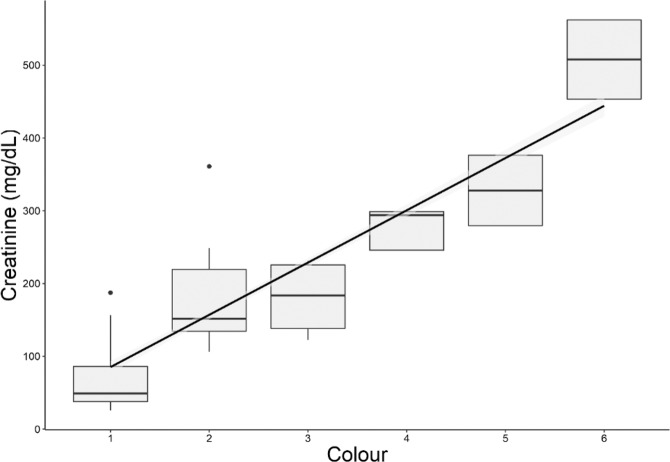
Validation of the methods used to measure hydration levels. Horizontal lines indicate the median. There is a positive correlation between the two methods (R2 = 0.7103).

Thirty-one urine samples from women with UTI were provided by Dr. Ana Cabrera from the Division of Microbiology at the Pathology and Laboratory Medicine Centre of London Health Sciences Centre (London, Ontario, Canada). All urine samples were obtained in accordance to article 2.4 of the Tri-Council Policy Statement: Ethical Conduct for Research Involving Humans – TCPS 2 (2018). All samples were provided anonymously for secondary use with ethics board approval not required. Samples were discarded after their primary use by Dr. Cabrera for standard clinical testing. These samples included 10 UTI-negative controls and 21 samples from patients clinically diagnosed with a UTI, from which 15 were positive for *E. coli*, 3 for *Staphylococcus aureus*, 2 for *Klebsiella pneumoniae*, and 1 for *Pseudomonas aeruginosa*. Diagnosis criteria were based on the presence of signs and symptoms and significant bacteriuria (bacterial growth ≥10^5^ colony-forming units (CFU)/mL). To further characterize the samples, pH was measured using test strips and creatinine was quantified. Figure 9 shows a linear correlation between both methods used to determine hydration levels.

For *in vitro* testing purposes, urine samples were collected for secondary use from three healthy pre-menopausal women and pooled for use in the experiment. All urine samples were obtained in accordance to article 2.4 of the Tri-Council Policy Statement: Ethical Conduct for Research Involving Humans – TCPS 2 (2018). All samples were provided anonymously for secondary use with ethics board approval not required. These were centrifuged at 4500 *g* for 10 min to remove any solid particles, followed by filter-sterilization using 0.22 µm syringe filters. To ensure asepsis, 10 µL of urine were plated on LB plates. To determine hydration levels, creatinine concentration was quantified using the Creatinine (urinary) Colorimetric Assay Kit (Cayman Chemical Co., Ann Arbor, MI, USA) as described by the manufacturer. Hydration levels were determined according to a validated 8-scale urine colour chart^44,45^.

### LC-MS analysis of urine samples

Samples were prepared based on the methodology proposed by Spagou *et al*.^46^. Briefly, aliquots of 250 µL were taken of each urine culture and diluted with pure methanol (1:3 for clinical samples and 1:6 for the *in vitro* analysis), vortexed, incubated on ice for 30 min, and centrifuged at x16000*g* for 10 min. Supernatants were then filtered using 0.22 µm PTFE syringe filters and added to vials for analysis. Samples were analyzed using a Q-Exactive Quadrupole Orbitrap MS, coupled to an Agilent 1290 HPLC system with an Agilent HILIC-Z (2.1 × 100 mm, 2.7 µm; Agilent) column. Compounds were resolved with mobile phases of 20 mM ammonium formate in water (A) and 20 mM ammonium formate in 90% acetonitrile (B) operating with the following gradient: 0 min, 100% B; 0.5 min, 100% B; 5.3 min, 80% B; 9.5 min, 30% B; 13.5 min, 30% B, 14.5 min 100% B and 16.5 min, 100% B. The following conditions were used for heated electrospray ionization (HESI): HESI(+) capillary voltage, capillary voltage, 3.5 kV; capillary temperature, 250 °C; sheath gas, 30.00 units; auxiliary gas, 8.00 units; probe heater temperature, 450 °C; S-Lens RF level, 60.00. The LC-MS was operated using a top 3 data-dependent acquisition (DDA) experiment that involved a full MS scan in the mass range of m/z 58–870 at 35,000 resolution, followed by MS/MS scans at 17,500 resolution, isolation window of m/z 1.2 and collision energy of 28. The biogenic amines and amino acids quantified in the full MS scans were: cadaverine, putrescine, tyramine, spermine, spermidine, histamine, TMA, phenylalanine, leucine, tryptophan, isoleucine, methionine, valine, tyrosine, proline, alanine, threonine, glutamine, serine, asparagine, glutamic acid, aspartic acid, histidine, arginine, cystine, lysine, ornithine, glycine, and TMAO. The standards used for calibration curves were acquired from Sigma Aldrich (St. Louis MO). TMAO was analyzed semi-quantitatively using the log transformation of its peak area (Figure S7).

### UPEC cultures

A time-course analysis was performed using clinical UPEC strains GR-12, IA2, 536 and J96, to assess whether there is a significant difference in polyamine production amongst strains over time. All the strains used were clinical isolates from patients with acute UTI^47–50^. Individual colonies of each strain were grown aerobically overnight in 5 mL of Lysogeny broth (LB) media, 5 µL of these cultures were then grown in sterile urine and incubated aerobically for 24 h at 37 °C. Control samples consisted of 5 mL of sterile urine with 5 µL of LB media; aliquots were taken at hours 0, 3, 6, 9, 12, and 24, and analyzed through HILIC-MS, post-acquisition recalibration was performed to the results, and the concentrations of the metabolites of interest was quantified. Growth was monitored measuring optic density at a wavelength of 600 nm to ensure uniform growth of the strains. Three biological replicates were used for each strain tested.

### LC-MS Data analysis

Proteowizard^51^ was used to convert the Thermo.raw files to.mzml format, with peak peaking filter applied. Features were detected using the XCMS package^52–54^ with the centWave^53^ method (ppm. Tolerance 1.0). The signal to noise threshold was set to 5, the noise was set to 3 × 10^6^ and pre-filter was set to six scans with a minimum 5,000 intensity. Retention time correction was conducted using the obiwarp^55^ method. Grouping of features was set to those present in at least 25% of all samples (retention time deviation 10 s; *m/z* width, 0.015). The ‘fillPeaks’ function with default settings. Remaining zeros values were imputed with two thirds the minimum value on a per mass basis. Large mass salt clusters and ionization artifacts were filtered using the McMillan correction^56^. For the targeted analysis, Xcalibur (Thermo Scientific, Waltham, MA) was used to quantify the metabolites of interest. Compounds were identified by accurate mass, comparison of retention times to authentic standards or by accurate mass and comparison of fragmentation patterns to MS/MS databases^57^.

The data generated were submitted to the EMBL-EBI MetaboLights database with the identifier MTBLS1294 (https://www.ebi.ac.uk/metabolights/MTBLS1294)^58^.

### Statistical analysis

RStudio version 1.2.1335^56^ was used for all statistical analyses. Plots were generated with the ggplot2 package^59^. For the untargeted metabolomics analysis, the ‘FactoMineR’^60^ package with Pareto scaling was used to perform a PCA. Targeted metabolomics comparisons in the clinical samples were analyzed using a two-sample two-tailed t-test analysis and defined a p-value of <0.05 as statistically significant. Time-course trials were analyzed using a linear mixed-effect model (Tukey post-hoc correction) using the ‘emmeans’ package^61^. Log transformation was used to correct the distribution and heteroscedasticity of the data^62^.

## Supplementary information

Supplementary information.

## Data Availability

The data generated were submitted to the EMBL-EBI MetaboLights database with the identifier MTBLS1294 (https://www.ebi.ac.uk/metabolights/MTBLS1294).
