# Supplementary material for: Malodorous biogenic amines in Escherichia coli-caused urinary tract infections in women—a metabolomics approach
Source: Sci Rep. 2020 Jun 16;10:9703. doi: 10.1038/s41598-020-66662-x (PMC7297715; doi:10.1038/s41598-020-66662-x)
Supplement: Supplementary file 1 — Supplementary information. [file 41598_2020_66662_MOESM1_ESM.docx]

**Malodorous biogenic amines in *Escherichia coli*-caused urinary tract infections in women—a metabolomics approach**

Scarlett Puebla-Barragan ^1,2^, Justin Renaud ^3^, Mark Sumarah^3^, and Gregor Reid ^1,2^*

^1^ Centre for Human Microbiome and Probiotics, Lawson Health Research Institute, London, ON, Canada

^2^  Departments of Microbiology & Immunology and Surgery, University of Western Ontario, London, ON, Canada

^3^ London Research and Development Centre, Agriculture and Agri-Food Canada, London, ON, Canada

***** Correspondence: gregor@uwo.ca; Tel.: +1-519-646-65256, ext. 65256

**
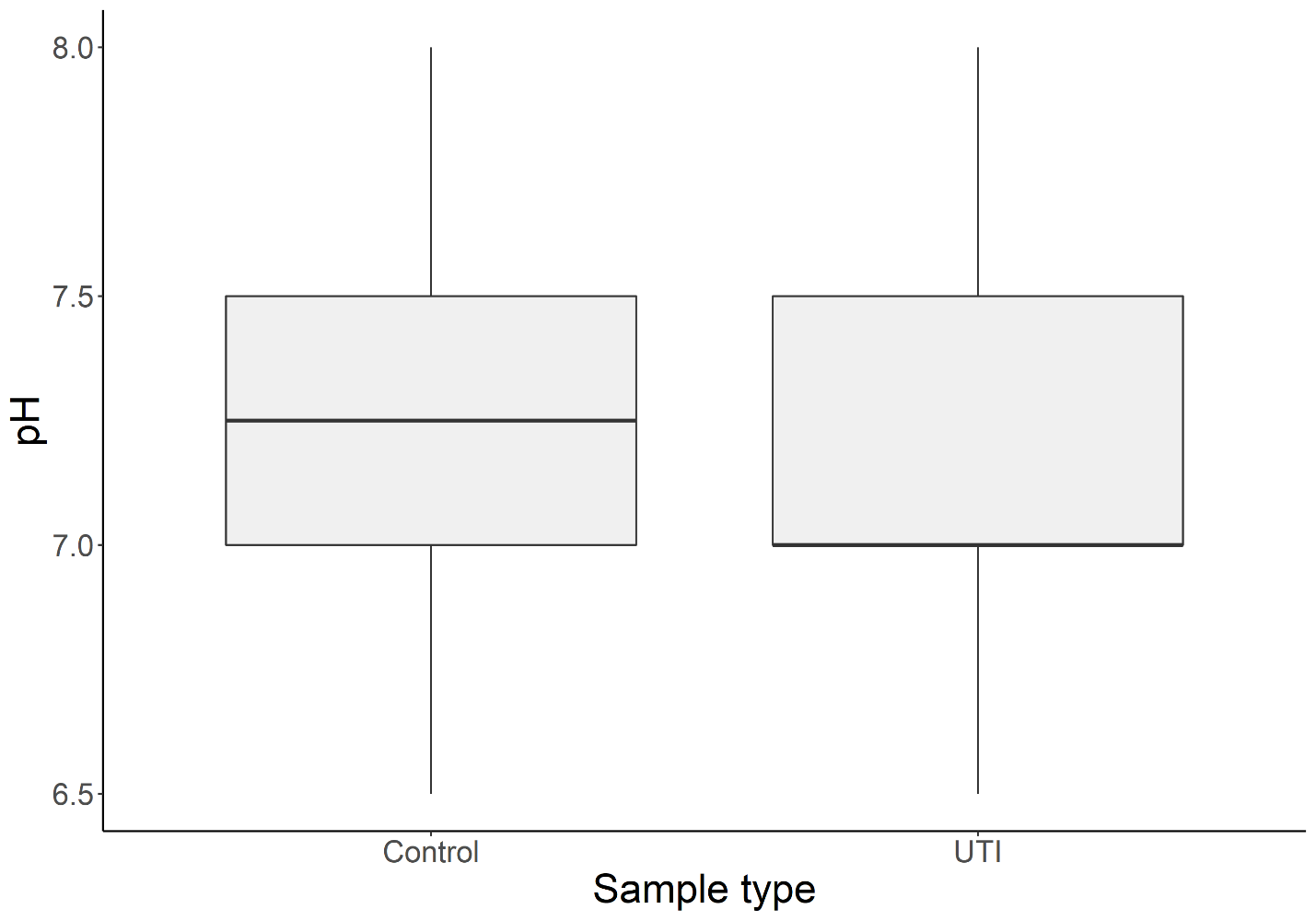
**

**Figure S1.** Comparison of pH values of Control vs UTI positive samples. Horizontal lines indicate the median. There was no significant difference between the groups. T-test analysis was used to calculate statistical significance.

**
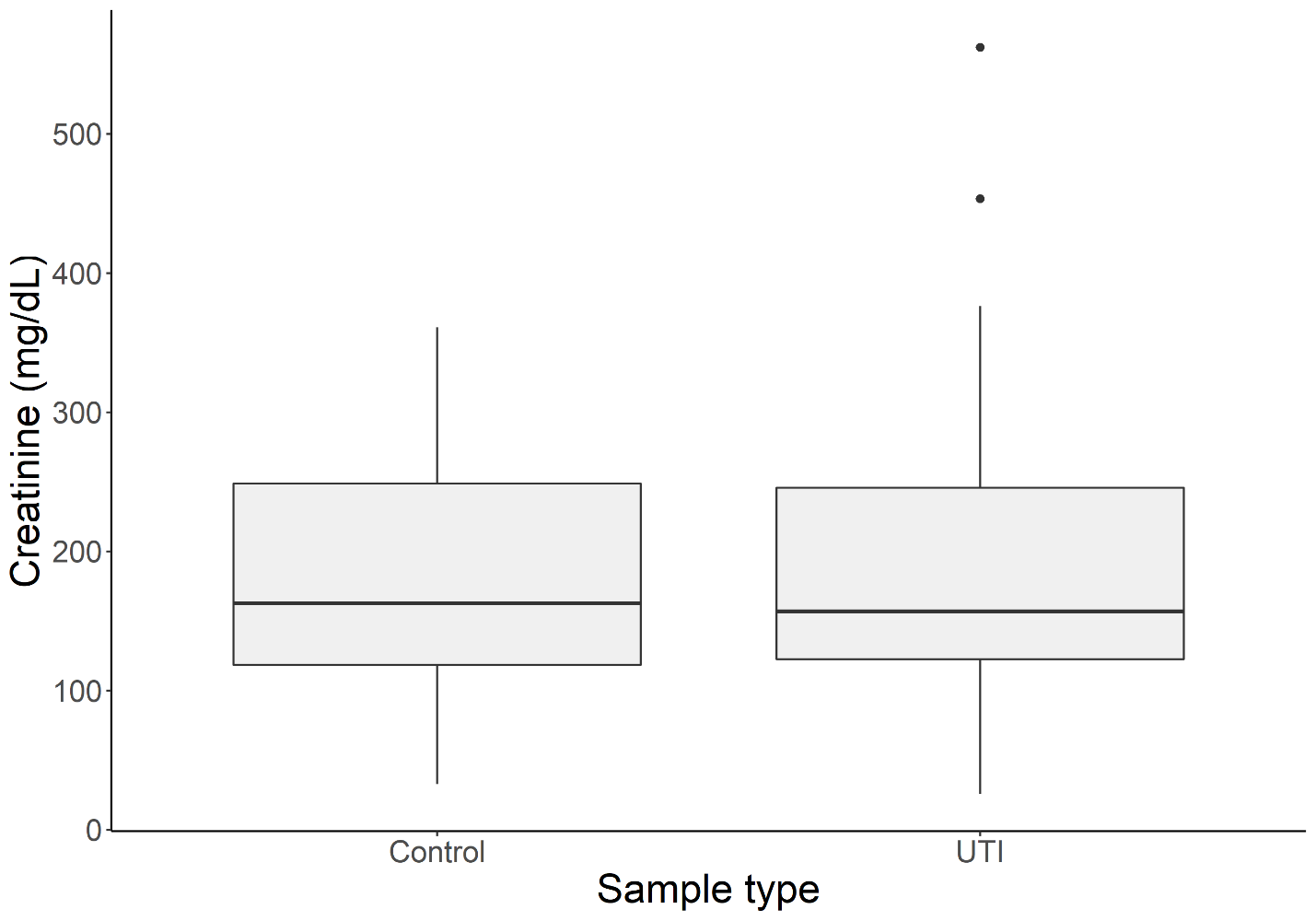
**

**Figure S2.** Comparison of creatinine values of Control vs UTI positive samples. Horizontal lines indicate the median. There was no significant difference between the groups. T-test analysis was used to calculate statistical significance.

**
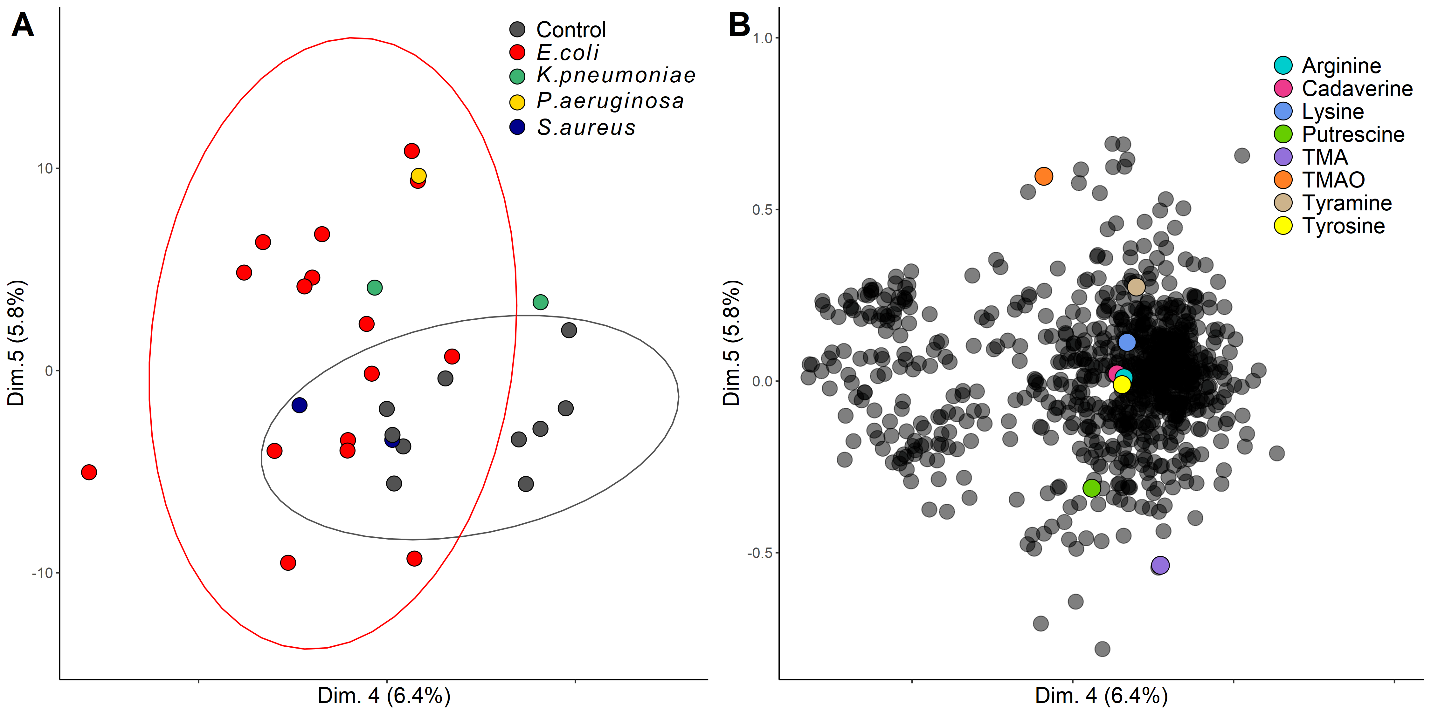
**

**Figure S3.** PCA analysis of samples including all pathogens**.** (A) PCA score plot of Healthy (black) vs UTI-positive samples (red), where each point represents a single sample from a single woman. The location of each point displays differences in the metabolome, with samples closer to each other being more similar. Ellipses represent the 95% confidence intervals; (B) PCA loadings, which show the weights of each metabolite in the principal component cartesian plane. Each point represents a single metabolite.

**
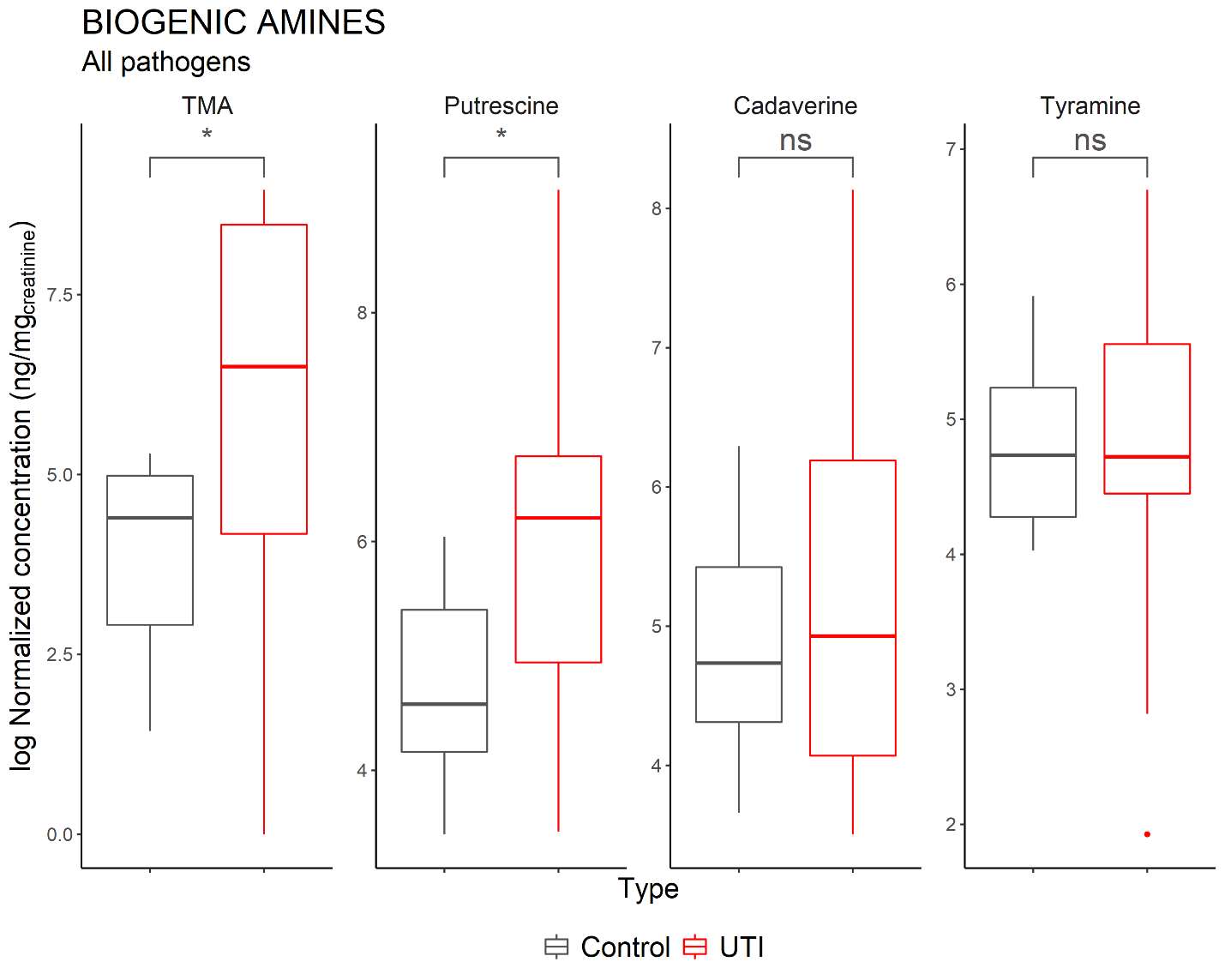
**

**Figure S4.** Comparison of the concentration of biogenic amines in Healthy vs UTI positive patients (all pathogens included**).** Control consists on urine of healthy patients. TMA and putrescine levels are elevated in patients with UTI. Only the samples positive for UPEC were included in this analysis. Horizontal lines indicate the median. Significant differences were determined on log-transformed values using a two-sample t-test (*p<0.05).

**
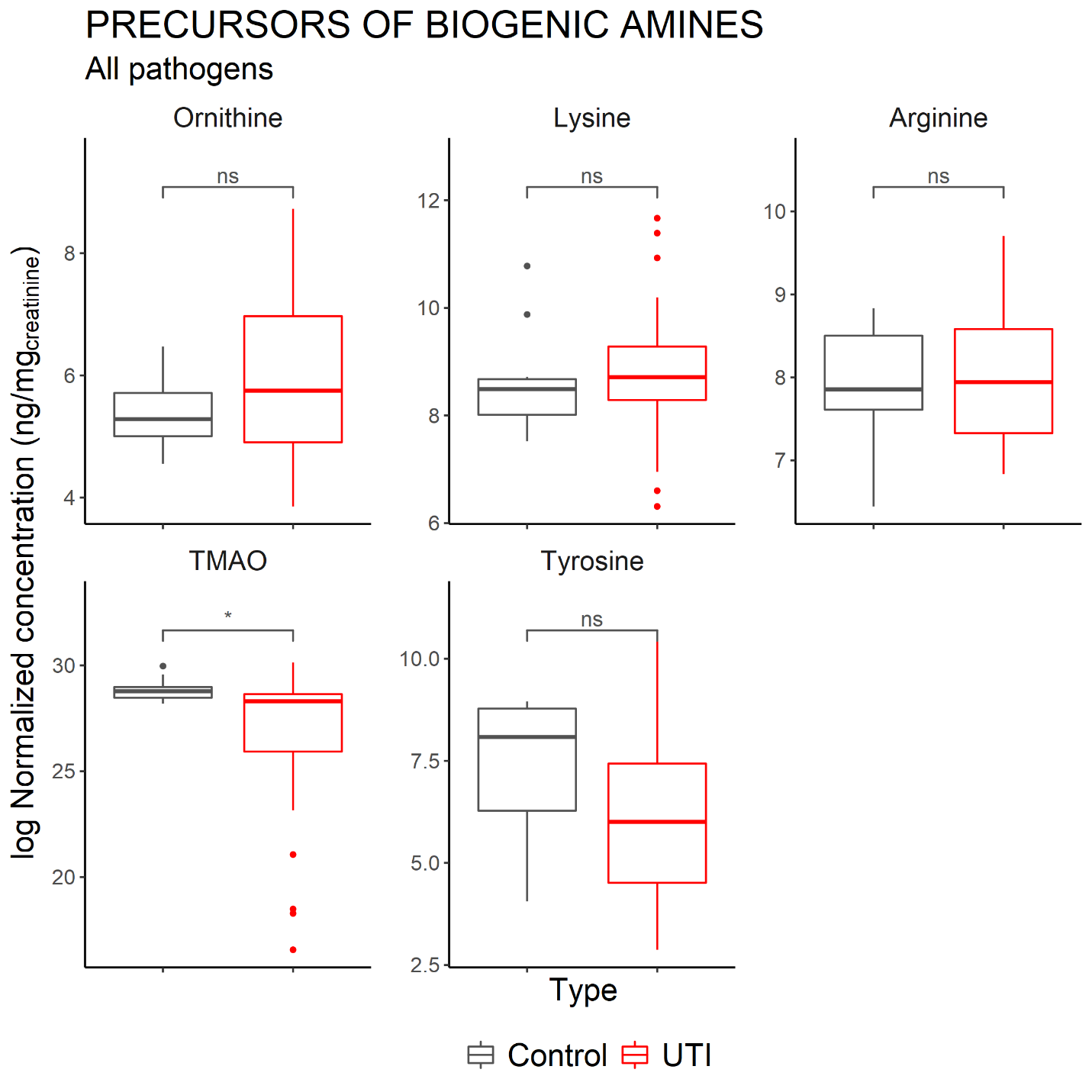
**

**Figure S5.** Comparison of the concentration of the precursors of biogenic amines in Healthy vs UTI positive patients (all pathogens included). Control consists on urine of healthy patients. Horizontal lines indicate the median. No significant differences were found between the groups. Statistical significance was determined on log-transformed values using a two-sample t-test (*p<0.05).

**
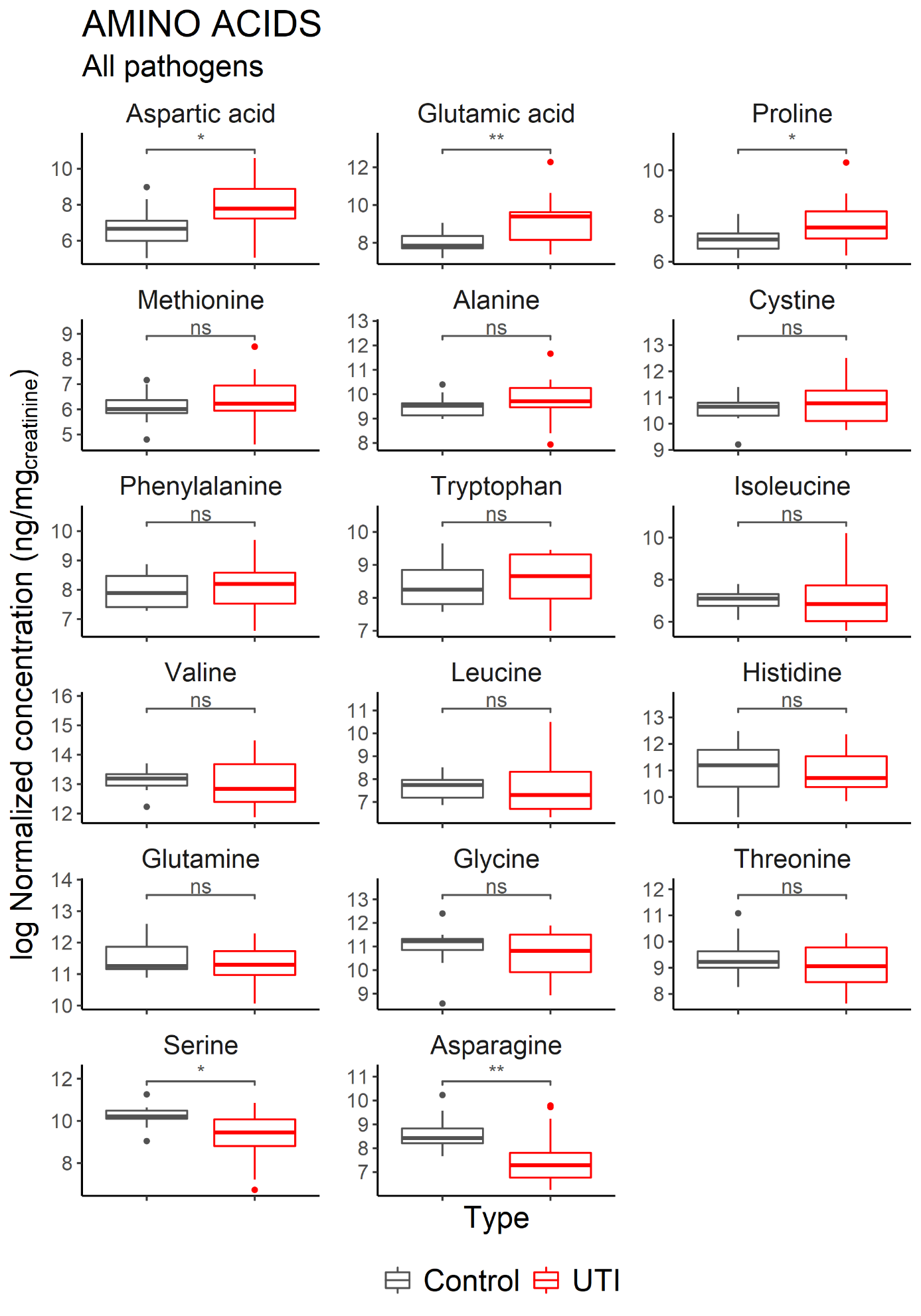
**

**Figure S6.** Comparison of the concentration of amino acids in Healthy vs UTI positive patients (all pathogens included). Control consists on urine of healthy patients. Glutamic acid and Aspartic acid are significantly elevated in UTI patients, while Serine and Asparagine are significantly decreased. Horizontal lines indicate the median. Significant differences were determined on log-transformed values using a two-sample t-test (*p<0.05, **p<0.005).

**Figure S7****.** LC-MS/MS spectra for TMAO.

**Table S1.** Multiple comparisons between strains of the concentration of biogenic amines across time (only significant values are shown, *p<0.05, **p<0.01, ***p<0.001, *****p<0.0001).

| Strain 1 | Metabolite | Time (h) | Strain 2 | p value | Significance level |
| --- | --- | --- | --- | --- | --- |
| 536 | Putrescine | 6 | IA2 | <0.0001 | **** |
|  |  | 12 | IA2 | 0.039 | * |
|  |  | 24 | IA2 | 0.0449 | * |
|  |  |  | J96 | 0.0449 | * |
|  | Cadaverine | 3 | GR-12 | <0.0001 | **** |
|  |  | 6 | GR-12 | <0.0001 | **** |
|  |  |  | IA2 | <0.0001 | **** |
|  |  |  | J96 | <0.0001 | **** |
|  |  | 9 | GR-12 | <0.0001 | **** |
|  |  |  | IA2 | <0.0001 | **** |
|  |  |  | J96 | <0.0001 | **** |
|  |  | 12 | GR-12 | <0.0001 | **** |
|  |  |  | IA2 | <0.0001 | **** |
|  |  |  | J96 | <0.0001 | **** |
|  |  | 24 | GR-12 | <0.0001 | **** |
|  |  |  | IA2 | <0.0001 | **** |
|  |  |  | J96 | <0.0001 | **** |
|  | TMA | 3 | GR-12 | <0.0001 | **** |
|  |  |  | IA2 | <0.0001 | **** |
|  |  | 6 | IA2 | <0.0001 | **** |
| GR-12 | Putrescine | 24 | IA2 | 0.0131 | * |
|  |  |  | J96 | 0.0131 | * |
|  | Cadaverine | 9 | IA2 | 0.0432 | * |
|  |  | 12 | IA2 | 0.0013 | ** |
|  |  | 24 | IA2 | 0.0001 | **** |
|  |  |  | J96 | 0.0113 | * |
|  | TMA | 3 | J96 | 0.0293 | * |
| IA2 | TMA | 3 | J96 | 0.0109 | ** |

**Table S2.** Multiple comparisons between strains of the concentration of amino acids and/or biogenic amines precursors across time (only significant values are shown, *p<0.05, **p<0.01, ***p<0.001, *****p<0.0001).

| Strain 1 | Metabolite | Time (h) | Strain 2 | p value | Significance level |
| --- | --- | --- | --- | --- | --- |
| 536 | TMAO | 3 | IA2 | <0.0001 | **** |
|  | Ornithine | 6 | GR-12 | 0.0058 | ** |
|  |  |  | J96 | <0.0001 | **** |
|  | Arginine | 3 | GR-12 | 0.0166 | * |
|  |  |  | IA2 | 0.002 | ** |
|  | Proline | 6 | J96 | 0.0014 | ** |
|  | Alanine | 3 | IA2 | 0.0355 | * |
|  |  | 6 | J96 | 0.0282 | * |
|  | Glutamic acid | 3 | IA2 | 0.0331 | * |
|  |  | 6 | GR-12 | 0.0013 | ** |
|  |  |  | IA2 | 0.0006 | *** |
|  |  |  | J96 | 0.0001 | **** |
|  |  | 9 | IA2 | 0.0014 | ** |
|  |  |  | J96 | 0.0007 | *** |
|  |  | 12 | GR-12 | 0.0013 | ** |
|  | Histidine | 3 | GR-12 | 0.0303 | * |
|  | Cystine | 6 | IA2 | 0.0002 | *** |
| GR-12 | TMAO | 3 | IA2 | 0.0187 | * |
|  | Tryptophan | 3 | IA2 | <0.0001 | **** |
|  | Glutamic acid | 3 | IA2 | 0.0283 | * |
|  |  | 12 | IA2 | 0.0031 | ** |
|  |  |  | J96 | 0.0406 | * |
| IA2 | Ornithine | 3 | J96 | 0.0385 | * |
|  |  | 6 | J96 | 0.0003 | *** |
|  | Tryptophan | 3 | IA2 | <0.0001 | **** |
|  | Proline | 6 | J96 | 0.001 | *** |
|  | Cystine | 6 | IA2 | 0.0115 | * |
| J96 | Asparagine | 3 | 536 | <0.0001 | **** |
|  |  |  | GR-12 | <0.0001 | **** |
|  |  |  | J96 | <0.0001 | **** |
|  |  | 6 | 536 | <0.0001 | **** |
|  |  |  | GR-12 | <0.0001 | **** |
|  |  |  | IA2 | <0.0001 | **** |
|  |  | 9 | 536 | 0.0242 | * |
|  |  |  | GR-12 | 0.0177 | * |
|  |  |  | IA2 | 0.016 | * |
|  |  | 12 | 536 | 0.005 | ** |
|  |  |  | GR-12 | 0.0163 | * |
|  |  |  | IA2 | 0.0074 | ** |
|  |  | 24 | 536 | 0.0004 | *** |
|  |  |  | GR-12 | 0.0006 | *** |
|  |  |  | IA2 | 0.0004 | *** |
